# Supplementary material for: Sensory Receptor, Inflammatory, and Apoptotic Protein Expression in the Bladder Urothelium of Patients with Different Subtypes of Interstitial Cystitis/Bladder Pain Syndrome
Source: Int J Mol Sci. 2023 Jan 3;24(1):820. doi: 10.3390/ijms24010820 (PMC9821243; doi:10.3390/ijms24010820)
Supplement: Supplementary file 1 [file ijms-24-00820-s001.zip › ijms-2087176-supplementary.pdf]

Figure S1. The scatter plots of the significant correlations betweem the expressions of (A,B) sensory receptors IC symptom scores, and expressions of inflammatory and pro-apoptotic proteins in IC/BPS patients; (C,D) sensorory receptors, inflammatory and pro-apoptotoc protein expressions and urodynamic paramters in IC/BPS patients; (E) Sensory receptors and urodynamic parameters.

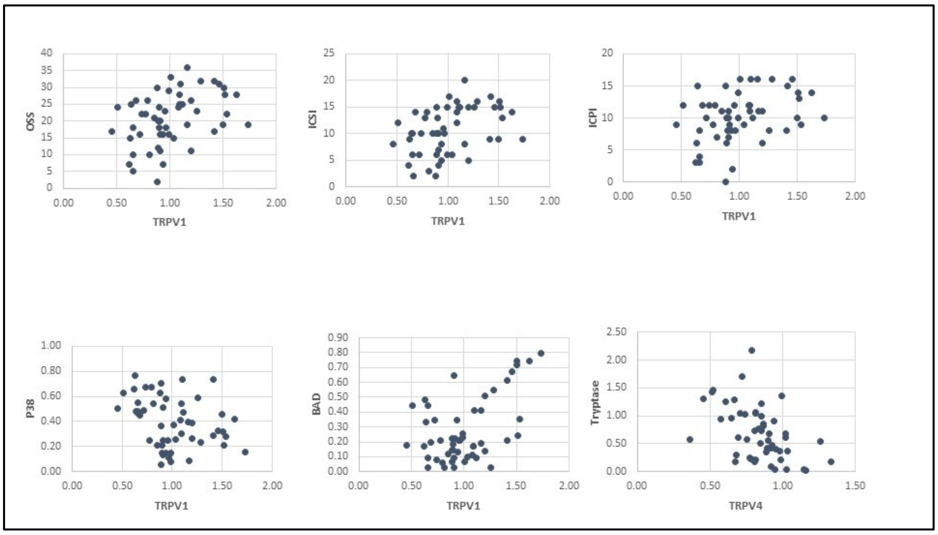

A

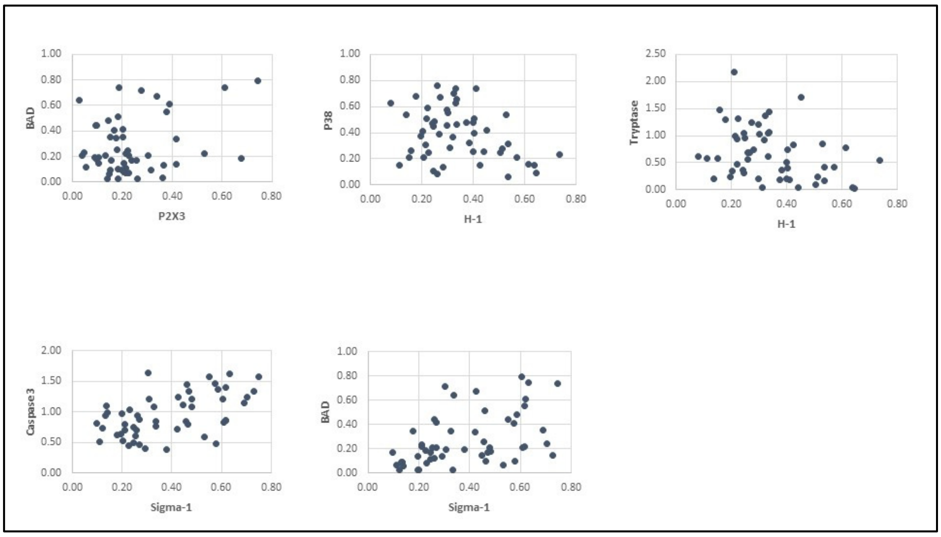

B

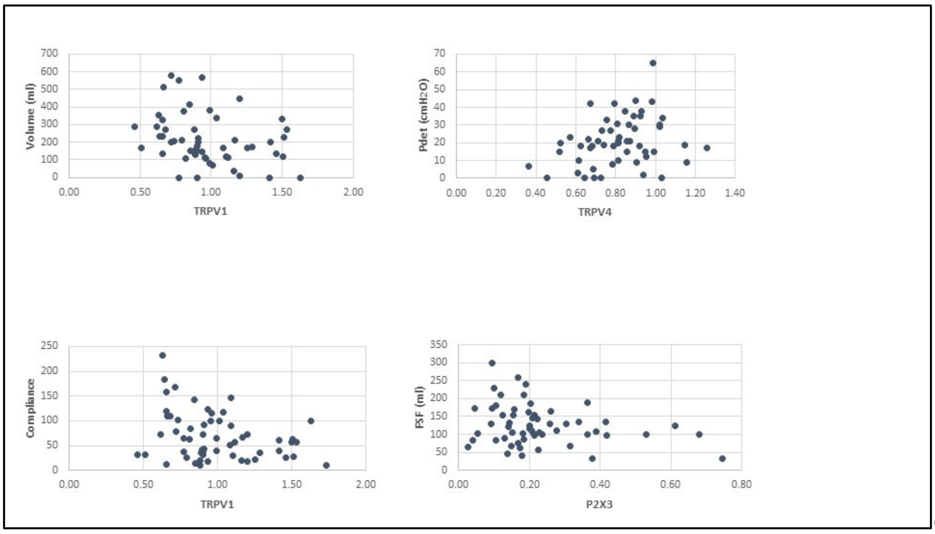

C

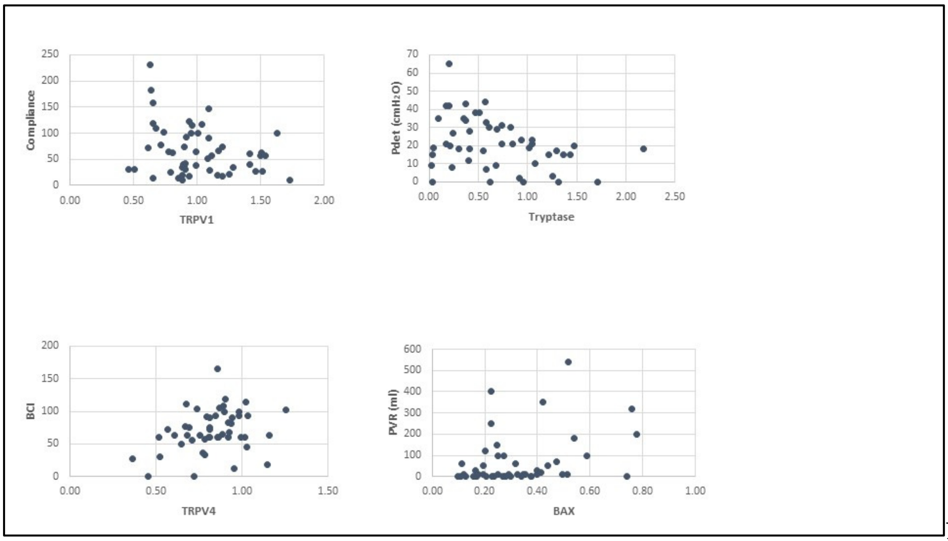
Compliance
TRPV1
0.00 0.50 1.00 1.50 2.00
0 50 100 150 200 250
Pdet (cmH2O)
Tryptase
0.00 0.50 1.00 1.50 2.00 2.50
0 10 20 30 40 50 60 70
BCI
TRPV4
0.00 0.50 1.00 1.50
0 50 100 150 200
PVR (ml)
BAX
0.00 0.20 0.40 0.60 0.80 1.00
0 100 200 300 400 500 600

D

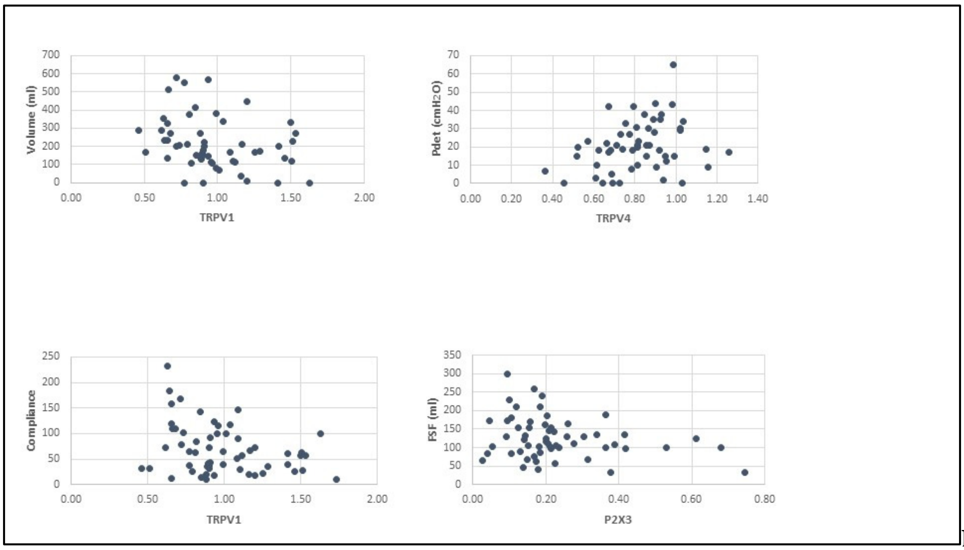
Volume (ml)
TRPV1
0.00 0.50 1.00 1.50 2.00
0 100 200 300 400 500 600 700
Pdet (cmH2O)
TRPV4
0.00 0.20 0.40 0.60 0.80 1.00 1.20 1.40
0 10 20 30 40 50 60 70
Compliance
TRPV1
0.00 0.50 1.00 1.50 2.00
0 50 100 150 200 250
FSF (ml)
P2X3
0.00 0.20 0.40 0.60 0.80
0 50 100 150 200 250 300 350

E

Table S1. The correlation between (A) bladder urothelium sensory receptors and urodynamic parameters in all IC/BPS patients and controls; (B) sensory receptors, inflammatory and pro-apoptotic protein expressions and the urodynamic parameters in IC/BPS patients.

(A) all IC/BPS + control	TRPA1	TRPV1	TRPV4	P2X3	H-1	Sigma-1
Pdet	-0.143	0.154	0.278*	-0.009	-0.022	0.004
Qmax	-0.024	-0.183	0.079	-0.022	0.079	-0.199
Volume	0.163	-0.294*	-0.104	-0.137	-0.110	-0.178
PVR	0.039	0.191	-0.015	-0.011	0.017	0.010
FSF	0.239	-0.241	-0.254	-0.261*	-0.146	0.023
FS	0.188	-0.153	-0.179	-0.205	-0.149	-0.134
Compliance	0.119	-0.313*	-0.219	-0.115	-0.149	-0.156
BCI	-0.081	-0.121	0.187	-0.030	0.064	-0.198
CBC	0.251	-0.074	-0.119	-0.171	-0.090	-0.137
cQmax	-0.075	-0.094	0.204	0.014	0.235	-0.103
VE	0.021	-0.261	0.045	-0.126	0.013	-0.044
AG	-0.086	0.232	0.149	0.011	-0.064	0.136

(B) IC/BPS	TRPA1	TRPV1	TRPV4	P2X3	H-1	Sigma-1	P38	Tryptase	Caspase3	BAX	BAD
MBC	0.124	0.075	-0.013	0.068	0.050	-0.196	0.040	0.056	-0.021	0.017	-0.033
Pdet	-0.156	0.138	0.261	-0.018	-0.028	-0.037	0.066	-0.354*	-0.126	-0.130	-0.142
Qmax	-0.102	-0.107	0.216	0.039	0.159	-0.089	0.144	0.009	0.068	0.124	-0.028
Vol	0.068	-0.215	0.066	-0.082	-0.025	0.014	0.243	0.014	-0.048	-0.058	-0.123
PVR	0.049	0.215	-0.027	0.010	0.027	0.009	0.032	0.172	0.151	0.356*	0.131
FSF	0.199	-0.182	-0.195	-0.235	-0.122	0.158	0.181	0.074	0.162	0.094	0.000
FS	0.121	-0.031	-0.056	-0.143	-0.088	0.052	0.194	0.117	0.123	0.107	0.065
Compliance	0.130	-0.280*	-0.162	-0.084	-0.126	-0.089	0.188	0.030	-0.032	0.055	0.029
BCI	-0.160	-0.051	0.310*	0.020	0.131	-0.109	0.154	-0.140	-0.001	0.058	-0.100
CBC	0.181	0.087	0.047	-0.090	0.020	0.086	0.219	0.101	0.091	0.280	0.096
cQmax	-0.101	-0.061	0.267	0.034	0.274	-0.056	-0.010	-0.139	0.092	0.155	0.060
VE	-0.004	-0.254	0.088	-0.132	0.027	-0.003	0.003	-0.219	-0.128	-0.164	-0.129
AG	-0.054	0.179	0.062	-0.033	-0.118	0.036	-0.036	-0.275	-0.131	-0.175	-0.080

Abbreviations: as in text, * p<0.05
